# A Criticism of the Editorial "Dental Faculties in Medical Schools."

**Published:** 1897-03

**Authors:** 


### Editorial, Etc.

### A CRITICISM OF THE EDITORIAL "DENTAL FACULTIES IN MEDICAL SCHOOLS."

The following is an editorial from the *Maryland Medical Journal* discussing a recent article, which is re-printed herewith.

### DENTAL AND MEDICAL SCHOOLS.

The *American Journal of Dental Science* objects to dental schools being appendages to medical schools, partly because the medical school, having part of the instructors already employed, can at a small additional cost obtain special men and equip a dental school, at much less cost than an independent school can be founded. Again the therapeutics of dentistry is unlike that of general medicine and a professor of materia medica and therapeutics in a medical school may be entirely unfit to teach his branch in a dental school. Added to this is the statement that sixty per cent. of the dental schools are connected with medical schools. That, it would seem, is an advantage.

The dentist has in time past, especially in this country, been considered a partly educated man. In no specialty of medicine in the United States does a man set out from the start to study that particular branch without taking up general medicine first, but the dentist, as a rule, gives attention only to the teeth and parts surrounding, and knows little of the rest of the body, so that it is hardly just to rank them with specialists. This does not apply so much to those dentists who have taken the degree of M. D., and that this is almost generally the custom in the large cities may be evidenced by the signs of leading dentists.

The desire for a general medical education is probably aroused by contact with the medical students in the schools to which these dental schools are attached. As a rule, to which there are some exceptions, the physician sets out in practice, taking any kind of case that presents itself, and he gradually drops into a specialty, and even then there are many specialists who, while paying particular attention to one branch, call themselves general practitioners and refuse nothing legitimate. There are also cases in which a young man, who has exceptional opportunities or who inherits a specialty, jumps in at once and skips the intermediate general practice stage, but it is likely that few dentists, even those with degrees of M. D., could treat a case or make a diagnosis outside of the mouth, hence the dentist does not, as a rule, rank with the broadly educated man as a specialist. This does not mean, of course, that one is not as much of a man as the other, for the dentist may far excel the physician in every other way.

While it may be advantageous for the dental branch of a school to have its own instructors in materia medica and and therapeutics, still the more the dentist knows of medicine the better it is for him, and if the day should ever come when the dentist thoroughly grasped medicine as an ordinary physician does and then took up dentistry as a specialty he would do much to raise the general standard of dentistry.

### DENTAL FACULTIES IN MEDICAL SCHOOLS.

There is a sentiment abroad that some dental schools have not the equipment of faculties and instructors that they should have. It is a prerequisite for admission to the National Association of Dental Faculties that the applicant shall have the endorsement of the Board of Dental Examiners of the State in which it is located; and it is the rule of the National Association of Dental Examiners that an application for recognition by that body must come from a member of the Association of Dental Faculties.

"Dental schools are not being formed now from an educational necessity. Two impulses now control this matter: first, personal ambition to have a position in and be connected with a dental school for the prominence it is supposed to give; and second, a purely commercial spirit on the part of medical schools. Over sixty per cent. of our schools are appendages to medical institutions; and nearly every dental school started in these later years has been under this outside influence."

The Committee on Colleges of the National Association of Dental Examiners recently expressed the view that more should be required to establish the right of dental schools to recognition than the fulfillment of the rules of the Association of Dental Faculties: evidence should be furnished that the teachers are of high standing, and that they and the school have the confidence of the local members of the profession. The committee also called attention of the impropriety of schools advertising as instructors practitioners who occasionally clinic before the students, but are not a part of the staff of the institution.

While "anybody with fair mechanical ability could pull or fill a tooth—and I have seen as good fillings as I ever saw inserted in teeth by a man wholly ignorant of the science of dentistry—and we all have seen the botches which educated men have made in attempting a similar operation," yet (in the words of an editorial in the January number of the Dental Cosmos) "the ability to teach dentistry is an acquisition quite distinct from, though supplemental to, the ability to practice it. So important has the art of teaching become that the study and investigation of its principles now constitute a distinct branch of scientific work, viz. pedagogics. To know dentistry from the point of view of the practitioner is one thing. To know dentistry from the teacher's standpoint implies not only all that is included in the educational equipment of the practitioner but in addition to this the important qualification of ability successfully to impart this knowledge to others."

Thr plan of requirements of dental schools hereafter making application for recognition by the National Association of Dental Examiners provides that each dental school must have a teaching faculty of at least three professors of dental subjects, namely, for operative dentistry, for dental prothesis, and for dental pathology and therapeutics; and at least five professors for medical subjects, namely, for anatomy, for physiology, for chemistry, for pathology and for materia medica.

Let us consider one thought in the discussion of "the equipment of faculties and instructors." In none of the dental departments of the medical schools known to us is there in the teaching faculty a professor for "dental pathology and therapeutics." The title used by faculties is "M. D. professor of materia medica and therapeutics" as certified to by official documents. Now, we maintain that more than a medical education (so called) is requisite to teach dental therapeutics. The therapeutics of dentistry, unlike its anatomy, physiology and pathology differs from that taught in the medical schools. It is medical, surgical and prosthetic. In so far as it is a direction of medical science to the prevention, modification or removal, by medicinal or hygienic remedies, of the causes and effects of disease in the dental organs it forms part of a physician's practice, just as does the treatment of cerebral, cardiac or pulmonary disease. In so far as it is an application of surgical skill to the extraction of teeth, the removal of tumors, to the treatment of fractures or of fissured palate it is simply oral surgery, involving only such knowledge and skill in the use of instruments as every surgeon must possess. But dental therapeutics includes a class of operations which medical men were not taught in medical schools, and which as physicians and surgeons, they do not practice. The prevailing and distinguishing feature of dental therapeutics is the art of replacement: replacement of dental *structure*, in such manner and with such material as shall prevent further action of the destructive agencies; replacement of dental *organs* by substitutes which shall physiologically restore impairment of function and æsthetically restore the natural expression of the face; and this, as was said before, a person with a medical education alone is not qualified to teach. R. G.

